# Maternal food literacy and health behaviors in relation to children’s DMFT scores: a cross-sectional study from Türkiye

**DOI:** 10.1186/s12903-025-07522-w

**Published:** 2025-12-19

**Authors:** Yavuzalp Solak, Gün Burak Tek, Hasan Durmuş, Erhan Kaya, Sevim Tek

**Affiliations:** 1 Çubuk District Health Directorate, Ankara, Turkey; 2 Private Dental Clinic, Osmaniye, Turkey; 3 Department of Public Health, Faculty of Medicine, Erciyes University, Kayseri, Turkey; 4 Department of Public Health, Faculty of Medicine, Kahramanmaraş Sutcu Imam University, Kahramanmaraş, Turkey

**Keywords:** Oral health, Food literacy, Health literacy, Dental caries, DMFT, Maternal behavior, Child health, Socioeconomic status

## Abstract

**Background:**

Oral health in childhood is influenced by parental literacy, dietary practices, and hygiene behaviors. Maternal food literacy may play a critical role in shaping children’s oral health, yet this relationship remains underexplored in low- and middle-income settings. This study aimed to investigate the association between maternal food literacy and the oral health status of their children, focusing on dental caries experience (DMFT scores), oral hygiene behaviors, and sociodemographic factors.

**Methods:**

A cross-sectional study was conducted in Dörtyol, Hatay (June 2022 – February 2023) involving 113 children aged 40–120 months and their mothers. Data were collected using validated Maternal Food Literacy and Maternal Health Literacy questionnaires, alongside structured questionnaires on children's oral health behaviors, while clinical oral examinations were performed to calculate DMFT (Decayed, Missing, Filled Teeth) scores. Statistical analyses included Spearman correlation, non-parametric tests, and multiple linear regression analysis using the backward elimination method, with p < 0.05 considered statistically significant.

**Results:**

The mean DMFT scores were 8.1 (±3.9) in children and 6.9 (±4.0) in mothers. While there was no significant relationship between the mother's food literacy and the child's DMFT scores (p=0.748), the mother's high health literacy was found to be associated with the child's high DMFT scores (B(95%CI) = 0.04 (0.01 – 0.08), p=0.025). Maternal education (p=0.019), employment status (p=0.003), and socioeconomic level (p=0.028) were significantly associated with DMFT. Behavioral factors—such as lack of personal toothbrush (p=0.018), low brushing frequency (p=0.002), and absence of fluoride varnish (p=0.008)—were also linked to higher caries levels. A modest but significant positive correlation was observed between maternal and child DMFT scores (r=0.264, p<0.01).

**Conclusion:**

Although maternal food literacy was not directly associated with children’s DMFT scores in bivariate analyses, the multiple linear regression model using the backward elimination method identified higher maternal health literacy as an independent predictor of increased caries experience in children. Socioeconomic conditions and oral health behaviors were also strongly associated with caries outcomes. These findings emphasize the importance of daily oral hygiene habits in children and suggest that oral health interventions should address behavioral education within families, regardless of literacy level.

## Introduction

Oral and dental health are key determinants of a child’s general health status and physical development, playing a crucial role in shaping their overall quality of life [1]. The family environment is widely recognized as a fundamental influence on child health, with parental health behaviors, knowledge, and health literacy emerging as critical factors affecting children’s physical and developmental well-being [2]. Notably, tooth decay stands out as the most common non-communicable disease worldwide, posing a significant public health challenge, especially in childhood. A global analysis by Wen et al. revealed that dental caries persisted as one of the most prevalent health conditions across all age groups from 1990 to 2019, with the highest occurrence of primary tooth decay observed in children aged 1 to 9 years [3]. A meta-analysis that included 28 studies conducted between 2000 and 2024 investigated the prevalence of dental caries in Turkish children and found a pooled prevalence rate of 75.6% [4]. The persistently high prevalence of dental caries indicates that it continues to be a largely overlooked yet pervasive public health concern. Despite regional disparities, its widespread distribution across Türkiye emphasizes the urgent need for early and effective preventive interventions. Therefore, it is essential to investigate social determinants—particularly parental health behaviors and knowledge—that shape children’s oral health outcomes [4].

Recent literature has increasingly focused on the role of parental factors, especially those of mothers, in shaping children’s oral health outcomes. A comprehensive systematic review by Firmino et al. reported a robust correlation between inadequate parental oral health literacy and elevated rates of dental caries among children [2]. However, comprehensive evidence regarding the relationship between parental oral health literacy and oral health indicators beyond dental caries remains limited. Nevertheless, studies indicate that children of parents with low oral health literacy tend to have higher caries scores, and parental awareness of their child’s dental treatment needs is directly associated with their level of oral health literacy [5]. Conversely, evidence suggests that parents’ awareness and perceptions of the significance of primary dentition are key determinants influencing both the oral health status of their children and their preferences concerning dental treatment options [6, 7]. While these studies consistently demonstrate a significant correlation between parental knowledge and children’s dental health outcomes, they also reveal persistent methodological and contextual limitations that hinder a comprehensive understanding of the underlying pathways and dynamics of this relationship [5]. Maternal health literacy is a crucial determinant of children’s overall health status, encompassing their oral health. Mothers who possess higher levels of health literacy tend to exhibit a better understanding of preventive care, adhere more effectively to dental recommendations, and actively encourage consistent toothbrushing and regular dental visits for their children [8, 9].

Childhood oral and dental health problems are largely driven by modifiable behavioral factors, such as feeding practices, frequency of sugar intake, and prolonged use of bottles or pacifiers. Among the determinants of these behaviors, maternal influence is particularly prominent, with maternal educational attainment and socioeconomic status closely linked not only to the child’s general health and developmental outcomes but also to parental engagement in preventive oral care and access to dental services [10, 11]. The consistently higher rates of dental caries observed among children of mothers with limited education and low socioeconomic status underscore the significance of this relationship. Consequently, health literacy, which encompasses individuals’ ability to obtain, process, and apply health-related information, has garnered substantial attention in recent years. Within this broader concept, oral health literacy has been increasingly recognized as a pivotal factor that shapes children’s oral hygiene practices, knowledge, and attitudes toward dental care [3]. The capacity of a mother to obtain, comprehend, and implement oral health information has the potential to significantly influence her child’s foundational oral hygiene behaviors, the nutritional quality of the child’s diet, and the regularity with which preventive dental services are accessed and utilized [2, 5].

Globally, research has explored various alternative oral hygiene interventions, including mouth rinses, xylitol-based products, and dental wipes, alongside conventional toothbrushing practices [3]. Nevertheless, the adoption of these alternative methods remains limited in Turkey, particularly within the pediatric population, primarily attributed to issues of accessibility and prevailing cultural norms [4]. Furthermore, emerging evidence underscores the critical role of food literacy and parental health literacy, particularly maternal health literacy, in influencing children’s dietary choices and oral hygiene practices across various stages of dentition. Specifically, studies consistently demonstrate a significant correlation between lower maternal health literacy levels and an elevated risk of early childhood caries, as well as suboptimal oral hygiene outcomes [10]. Food literacy encompasses the competencies required to plan, acquire, and prepare food that promotes overall health. Within the domain of oral health, insufficient food literacy can lead to behaviors such as the frequent consumption of sugary snacks, irregular meal patterns, and a limited understanding of cariogenic foods, all of which are recognized determinants of dental caries in children. Thus, food literacy may function as a behavioral intermediary linking maternal educational background to children’s oral health outcomes [12, 13].

While health literacy has been extensively studied in relation to oral health, the role of food literacy, despite its direct relevance to dietary behaviors, has been largely neglected. Therefore, exploring the impact of maternal food literacy on children’s oral health is a critical and underexplored area. This study aims to investigate the associations between mothers’ health and food literacy competencies and their children’s oral and dental health indicators, with a specific focus on the predictive value of these literacy domains in shaping oral health outcomes. It was hypothesized that increased maternal food literacy would correlate with lower Decayed, Missing, and Filled Teeth scores in children, with this association being potentially influenced by oral hygiene behaviors and sociodemographic factors.

## Methods

This cross-sectional study took place in Dörtyol, Hatay, from June 2022 to February 2023. Researchers collected data from children visiting a dental clinic with their mothers, gathering dental examination results and conducting in-person interviews with the mothers. Inclusion criteria required that participants be biological mothers of children aged 36–120 months. Children with diagnosed systemic diseases, developmental disabilities, or ongoing orthodontic treatment were excluded from the study.

The sample size was calculated using G*Power version 3.1.9.2, assuming a moderate effect (f = 0.3) size for the relationship between mothers’ food literacy and health literacy levels and their children’s oral and dental health outcomes. A two-tailed hypothesis was established, with a significance level of α = 0.05 and a power of β = 0.90, using the correlation–bivariate normal model test, which determined the required sample size to be 112 participants.

**Fig. 1:**
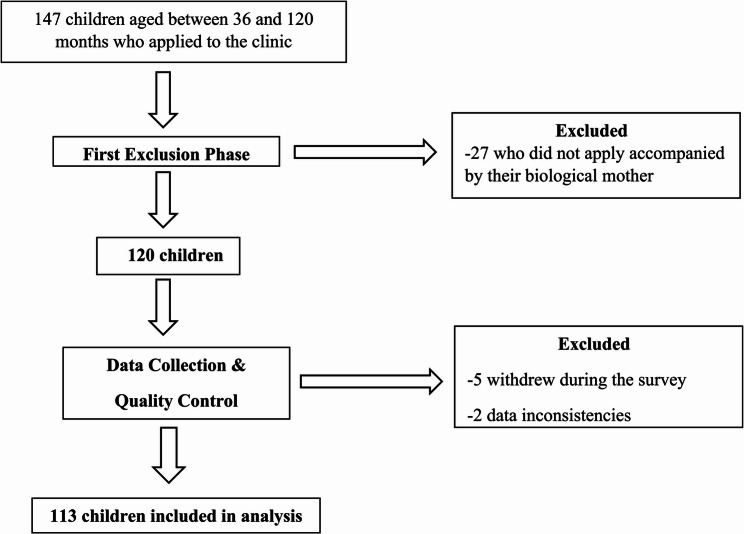
Participant inclusion, exclusion, and final sample numbers of study

To account for possible missing or erroneous data, 147 participants were included in the study at the outset. However, 27 children who did not attend the clinic with their biological mothers were excluded from the study, five of the remaining 120 participants withdrew during the survey, and two were excluded due to data inconsistencies. This left a final sample size of 113 participants for analysis. Inclusion, exclusion, and participant numbers are shown in Fig. 1.

The survey consisted of three sections. The first section included items related to sociodemographic characteristics, children’s dietary habits, and tooth brushing routines, as well as the DMFT (Decayed, Missing, and Filled Teeth) scores for both the mother and the child, and dmft scores for childs deciduous teeth. Which were completed by the clinic dentist.

Given that the study population included children in both the primary and mixed dentition stages (40–120 months of age), dental caries experience was assessed using the dmft index for primary teeth and the DMFT index for permanent teeth. Because this age range encompasses varying stages of tooth eruption, a combined caries experience score (dmft + DMFT) was used to represent the total burden of dental caries, while acknowledging potential differences in dentition status among participants. The total caries experience was calculated as the sum of these two indices, providing a single composite measure for each participant. This approach allowed for a more comprehensive assessment of the total caries burden in children transitioning from primary to permanent dentition. DMFT and dmft scores were obtained through separate clinical examinations conducted by two dentists. In cases of inter-rater discrepancies, a third dentist was consulted to reach a consensus. Although interrater reliability was not formally calculated, all examiners were calibrated prior to data collection using WHO diagnostic criteria. All oral health and feeding behavior data were obtained via structured self-administered questionnaires completed by the mothers. Socioeconomic Status (SES) was assessed using two self-reported indicators: maternal education level and monthly household income. Participants were grouped into low, moderate, or high SES categories based on national income percentiles.

In the second section, the 12-item Short Food Literacy Questionnaire, originally developed by Krause [14] and adapted and validated for the Turkish population by Durmuş [15] was administered to assess participants’ food literacy levels. Participants can obtain scores ranging from 7 to 52 on the Food Literacy Scale, with higher scores reflecting greater levels of food literacy. A score of 31 or above is considered indicative of adequate (good) food literacy, whereas scores below 31 reflect inadequate (poor) food literacy [16]. In the third section, participants completed the 32-item Health Literacy Scale derived from the European Health Literacy Survey Questionnaire (HLS-EU), for which the Turkish version was validated and deemed reliable by Okyay [17]. The scale is based on a five-point Likert-type format, with response options including ‘very easy,’ ‘easy,’ ‘difficult,’ ‘very difficult,’ and ‘I have no idea.’ Scores on the scale range from 0 to 50, with higher scores reflecting greater levels of health literacy. The internal consistency reliability (Cronbach’s α) of the Food Literacy Scale in our sample was 0.86, and for the Health Literacy Scale 0.81. These values are consistent with those reported in the original Turkish validation studies (respectively 0.88 and 0.83).

Statistical analyses were performed using SPSS 22.0 for Windows. Descriptive statistics, including frequency and percentage distributions, were used to summarize categorical variables. Continuous variables with a normal distribution were presented as mean ± standard deviation, while non-normally distributed variables were presented as median. The Kolmogorov–Smirnov test was used to assess the normality of data distribution. For quantitative data analysis between independent groups, Mann–Whitney U tests were used for non-normally distributed data. For comparisons involving more than two groups, the Kruskal–Wallis test was employed, followed by Tamhane’s T2 post hoc analysis when appropriate. Statistical significance was set at *p* < 0.05. Correlation analyses were conducted using the Spearman correlation test. Descriptive statistics were computed to summarize the participants’ sociodemographic and oral health–related characteristics. Prior to the regression analysis, the assumptions of normality, linearity, homoscedasticity, and multicollinearity were examined and found to be within acceptable limits. To identify the predictors of the child’s DMFT score, a multiple linear regression analysis using the backward elimination method was performed. In this procedure, all potential independent variables, including sociodemographic factors, parental education levels, oral health behaviors, nutritional indicators, parental DMFT, and total social literacy score, as well as significant dichotomous variables and theoretically relevant potential predictors were initially entered into the model. Variables with a p value greater than 0.10 were sequentially removed in a stepwise manner until the final model containing only statistically significant predictors remained. The level of significance was set at *p* < 0.05.In this study, maternal food and health literacy were treated as independent variables, and children’s DMFT scores as the primary outcome (dependent variable). Sociodemographic characteristics and oral hygiene behaviors were considered as potential influencing factors.

The study was conducted in accordance with the ethical principles outlined in the Declaration of Helsinki, and ethical approval was obtained from the Ethics Committee of Hatay Mustafa Kemal University (Date: 22.04.2021, Decision No: 09).

## Results

A total of 113 children (52.2% girls) participated in the study. The children’s ages ranged from 40 to 120 months, with a median of 73 months, and their mean BMI was 17.0 ± 2.6. The median DMFT score was 8.0 (0–18) (mean ± standart deviation = 8.1 ± 3.9) for children and 6.0 (0–18) (mean ± standart deviation = 6.9 ± 4.0) for mothers. The average age of mothers was 34.3 ± 4.5 years, and that of fathers was 37.8 ± 4.7 years. Educational attainment among mothers was mostly at high school (36.3%) and university (38.1%) levels, while fathers predominantly had university (42.5%) and postgraduate (17.7%) education. While 41.6% of mothers were employed, 99.1% of fathers reported working. Most mothers rated their economic status as moderate (57.5%) or good (34.5%). Regarding oral health behaviors, 80.5% of children had their own toothbrush, but only 18.6% brushed twice daily, and 16.8% did not brush at all. Among mothers, 54.9% brushed their teeth twice daily. Only 29.2% of children had never visited a dentist for check-ups, and 26.5% of mothers reported taking their child to regular dental check-ups even in the absence of complaints. Early-life nutrition data showed that 61.1% of children were exclusively breastfed for the first 6 months, with a median breastfeeding duration of 15 months. The median introduction to complementary foods was 6 months, and 38.9% began before 6 months. Bottle and pacifier use durations had a median of 12 months. Nearly half of the families (46.0%) had another household member with dental problems. The frequency of cariogenic food consumption varied, with 27.4% consuming such foods several times daily. Only 20.4% of children had received fluoride varnish application through public health services, while 72.6% had never received it. Mothers’ mean food literacy score was 35.8 ± 5.3, with 83.2% classified as having good food literacy. The mean health literacy score was 35.9 ± 7.9; 71.7% of mothers demonstrated sufficient or excellent health literacy.

When examining the factors associated with children’s DMFT scores, no statistically significant differences were observed based on gender, age group, or the presence of siblings. Similarly, variables such as birth timing, frequency of illness requiring medication, and the presence of a chronically ill family member did not show significant associations with DMFT scores. However, statistically significant differences were identified in relation to several sociodemographic and behavioral variables: Maternal education was significantly associated with DMFT scores; specifically, children of mothers with a high school education or less exhibited higher scores compared to those whose mothers had a university education or higher (Z = −2.346, *p* = 0.019). Maternal employment status also demonstrated a significant relationship, with children of unemployed mothers displaying higher DMFT scores than those of employed mothers (Z = −2.986, *p* = 0.003). Paternal education level was a significant factor; children of fathers with a high school education or less had higher DMFT scores than those whose fathers had a university education or higher (Z = −1.996, *p* = 0.046). Children from families with moderate or poor perceived economic status had significantly higher DMFT scores than those from families with good or very good economic status (Z = −2.198, *p* = 0.028). Regarding infant feeding practices, children who were exclusively breastfed for the first six months showed higher DMFT scores than those who received complementary feeding within the same period (Z = −2.147, *p* = 0.032). These findings underscore the significant influence of parental education, employment status, socioeconomic conditions, and early nutrition on children’s dental health. Children who received complementary feeding during the first six months had higher DMFT scores compared to those who received complementary feeding during the same period (Z = −2.147, *p* = 0.032) (Table 1).

**Table 1:** Sosyodemographic factors associated with children’s DMFT scores

	*n*	Childrens DMFT Score Median (Min-max)	Test *p*
Gender			Z
Female	59	8.0(0.0–16.0)	−1.562
Male	54	8.0(1.0–18.0)	0.118
Age			
Preschool	54	8.0(0–16.0)	−1.190
School	59	8.0(0–18.0)	0.234
Sibling			
No	29	9.0(0–16.0)	−1.102
Yes	84	8.0(0–18.0)	0.270
Maternal Education			
High school and less	57	8.0(0–18.0)^a^	**−2.346**
University and high	56	8.0(0–15.0)^b^	**0.019**
Maternal Employment			
Employed	47	8.0(0–15.0)^a^	**−2.986**
Unemployed	66	9.0(0–18.0)^b^	**0.003**
Paternal Education			
High school and less	45	8.0(1.0–18.0)^a^	**−1.996**
University and high	68	8.0(0–15.0)^b^	**0.046**
Family Economic Status			
Moderate and poor	66	8.0(0–18.0)^a^	**−2.198**
Good and very good	47	8.0(0–16.0)^b^	**0.028**
Time of birth			
Premature < 37 week	11	8.0(0-18.0)	5.277
On Time 37–42 week	96	8.0(0–16.0)	0.071
Postmature > 42 week	6	5.5(1.0–8.0)	
Breastfeeding			
Exclusively breastfed	69	8.0(0–18.0)^a^	**−2.147**
Complementary feeding	44	8.0(0–16.0)^b^	**0.032**
Having medication require illness			
Once a month or more	24	8.5(0–16.0)	0.592
Several times a year	67	8.0(0–16.0)	0.744
Once a year or less	22	8.0(0–18.0)	
Family member with chronic disease			
Yes	10	8.0(3.0–12.0)	−0.214
No	103	8.0(0–18.0)	0.830

Mann Whitney-U test was applied in the analysis of 2 variables, Kruskall Wallis test was applied in the analysis of more than 2 variables, and posthoctamhane’s T2 analysis was applied. There is a significant difference between the values indicated by the letters a and b

A significant difference was observed in children’s DMFT scores based on toothbrush ownership; children without a personal toothbrush exhibited significantly higher DMFT scores compared to those who did (Z = −2.358, *p* = 0.018). Toothbrushing frequency also had a statistically significant effect on DMFT scores (*p* = 0.002); children who brushed less than once per day, or not at all, had significantly higher DMFT scores than those who brushed once or twice daily. However, no significant differences were observed in DMFT scores based on whether the child had previously visited a dentist for a check-up (*p* = 0.481), whether they were regularly taken to the dentist even in the absence of problems (*p* = 0.344), whether the mother regularly attended dental check-ups herself (*p* = 0.318), or the frequency of cariogenic snack consumption (*p* = 0.718). In terms of milk consumption, children who drank milk several times per week had significantly lower DMFT scores compared to those who consumed milk less frequently (Z = −2.116, *p* = 0.034). Children who had received a fluoride varnish application had significantly lower DMFT scores than those who had not (Z = −2.639, *p* = 0.008). No statistically significant association was found between maternal food literacy level and children’s DMFT scores (*p* = 0.748), and although differences were observed among groups based on maternal health literacy levels, these differences did not reach statistical significance (*p* = 0.076) (Table 2).

**Table 2:** Maternal and child health-related factors associated with children’s DMFT scores

	*n*	Childrens DMFT Score Median (Min-max)	Test *p*
Child’s own toothbrush			
Own	91	8.0(0–18.0)^a^	**−2.358**
Does not have	22	9.5(4.0–16.0)^b^	**0.018**
How often the child brush teeth?			
Not brushing	19	10.0(4.0–16.0)^b^	**14.369**
Less than once a day	20	9.0(5.0–15.0)	**0.002**
Brushing once a day	53	8.0(0–18.0)^a^	
Brushing twice a day	21	8.0(0–15.0)^a^	
Has the child does a dental checkup			
Yes	80	8.0(0–16.0)	−0.705
No	33	8.0(0–18.0)	0.481
Regular dental check-up			
Yes	28	8.0(0–15.0)	−0.947
No	85	8.0(0–18.0)	0.344
Mothers regular dental check-up			
Yes	30	8.0(0–14.0)	−0.998
No	83	8.0(0–18.0)	0.318
Decay-causing junk food, sugar, etc.			
Consumed daily	45	8.0(0–18.0)	−0.361
Consumed less frequently	68	8.0(0–16.0)	0.718
How often does child drink milk?			
A few times a week	67	8.0(0–16.0)^a^	**−2.116**
Less than once a week or never	46	9.0(0–18.0)^b^	**0.034**
Fluoride varnish			
Made	31	8.0(0–15.0)^a^	**−2.639**
Not Made	82	8.0(0–18.0)^b^	**0.008**
Maternal Food Literacy Level			
Good food literacy	94	8.0(0–16.0)	−0.322
Bad food literacy	19	9.0(0–18.0)	0.748
Maternal Health Literacy Level			
Inadequate	12	8.0(0–18.0)	6.871
Problematic/Limited	20	8.0(0–12.0)	0.076
Sufficient	59	9.0(0–16.0)	
Excelent	22	8.0(0–14.0)	

Mann Whitney-U test was applied in the analysis of 2 variables, Kruskall Wallis test was applied in the analysis of more than 2 variables, and PosthocTamhane’s T2 analysis was applied. There is a significant difference between the values indicated by the letters a and b

A statistically significant positive correlation was observed between children’s and mothers’ DMFT scores (*r* = 0.264, *p* < 0.01), suggesting that higher maternal caries experience is associated with increased caries levels in children. Significant positive correlations were also found between children’s DMFT scores and both pacifier use duration (*r* = 0.508, *p* < 0.01) and bottle-feeding duration (*r* = 0.385, *p* < 0.01), indicating that prolonged use of these items may elevate caries risk. Children’s age did not significantly correlate with DMFT scores but showed a positive correlation with maternal age (*r* = 0.356, *p* < 0.01). Exclusive breastfeeding duration was negatively correlated with child age (*r* = −0.206, *p* < 0.05), reflecting a trend toward shorter breastfeeding in older children. Maternal food literacy was negatively correlated with child age (*r* = −0.212, *p* < 0.05), and maternal health literacy exhibited a stronger negative correlation (*r* = −0.326, *p* < 0.01), suggesting potentially higher literacy levels among younger mothers.

Maternal food literacy and health literacy scores displayed a strong positive correlation (*r* = 0.536, *p* < 0.01), demonstrating a close relationship between these competencies. Furthermore, a strong positive correlation was noted between pacifier and bottle use durations (*r* = 0.625, *p* < 0.01), indicative of concurrent feeding habits. Other variables, including the timing of the first antibiotic use, age at introduction to complementary feeding, and breastfeeding duration, showed weak or no significant associations with DMFT scores (Table 3).

**Table 3:** Association of various maternal and child variables with DMFT scores

	Child DMFT	Maternal DMFT	Child Age (month)	Maternal Age (year)	Childs Breastfeed Duration	Introduction to Complementary Feeding	Childs First Antibiotic Use age	Childs Pacifier Use Duration	Childs Bottle Use Duration	Maternal Food Literacy Score	Maternal Health Literacy Score
Chil DMFT	1										
Maternal DMFT	0.264^**^	1									
Child Age (Month)	0.078	0.355^**^	1								
Maternal Age (Year)	−0.010	0.104	0,356^**^	1							
Breastfeed Duration	−0.072	−0,051	−0.206^*^	−0.121	1						
Introduction to Complementary Feeding	0.104	0,061	0,066	−0.040	0,326^**^	1					
First Antibiotic Use Age	0.044	0.042	−0.106	−0.112	0.011	0.193^*^	1				
Pacifier Use Duration	0.508^**^	0.022	0.093	0.084	−0.050	0.086	0.015	1			
Bottle Use Duration	0.385^**^	0.072	0.137	0.081	0.021	0.154	0.067	0.625^**^	1		
Maternal Food Literacy Score	−0.026	−0.022	−0.212^*^	0.025	0.104	−0.140	−0.015	−0.086	0.026	1	
Maternal Health Literacy Score	0.021	−0.093	−0.326^**^	−0.126	0.122	−0.119	0.061	−0.177	−0.082	0.536^**^	1

*: p<0.05, **: p<0.01

The backward multiple linear regression model accounted for 52% of the observed variance in children’s DMFT scores. Specifically, the analysis revealed that male children exhibited significantly higher DMFT scores compared to their female counterparts. Conversely, lower paternal education levels and exclusive breastfeeding practices were independently associated with reduced DMFT scores. Furthermore, the absence of a personal toothbrush and prolonged pacifier use were identified as significant predictors of elevated DMFT values. Additionally, both higher maternal DMFT scores and greater maternal health literacy scores emerged as significant predictors of increased DMFT scores in their offspring. Collectively, these findings underscore the critical influence of both behavioral and familial determinants, specifically oral hygiene practices, feeding patterns, and maternal oral health status, in modulating the dental caries experience among children (Table 4).

**Table 4:** Multiple linear regression model identifying predictors of children’s DMFT scores

Factor	B (95% Cl)	St. Er.	T	*p*
Constant	−2.39 (−6.84–2.05)	2.24	−1.07	0.287
Gender	1.18 (0.48–2.32)	0.57	2.06	0.041
Paternal Education	−1.58 (−2.75 – −0.41)	0.59	−2.68	0.009
Breastfeeding	−1.55 (−2.69 – −0.41)	0.57	−2.70	0.008
Child’s own toothbrush	1.50 (0.39–2.95)	0.73	2.03	0.045
Pacifier Use Duration	0.28 (0.20–0.36)	0.04	6.75	< 0.001
Maternal DMFT	0.22 (0.09–0.36)	0.07	3.25	0.002
Maternal Health Literacy Score	0.04 (0.01–0.08)	0.02	2.28	0.025

R^2^: 0.52

## Discussion

In our study, which included a total of 113 children aged between 40 and 120 months, 52.2% of whom were girls, the mean DMFT score was found to be 8.1. In a study conducted at the Faculty of Dentistry in Istanbul, the DMFT scores were reported as 6.05 for boys and 5.84 for girls [18]. In Indonesia, the mean DMFT score was 3.42 among elementary school students and 3.79 among middle school students [19]. Overall, the mean DMFT scores observed in our study population were relatively high. This may be attributed to the inclusion of both primary and mixed dentition periods in our sample, resulting in the combined assessment of DMFT and dmft indices. Therefore, potential factors contributing to these elevated scores should be investigated in greater depth.

In this study children who were exclusively breastfed during the first six months showed significantly higher DMFT scores, and a positive correlation was identified between the duration of pacifier and bottle use and DMFT scores. In a study by Shahrabi et al. [20], dental caries were evaluated in relation to maternal age, breastfeeding duration, consumption of sweetened beverages via bottle, and the age at which toothbrushing began. The authors concluded that longer breastfeeding duration and delayed initiation of toothbrushing were significantly associated with a higher prevalence of caries. Sanders et al. reported similar findings, linking prolonged breastfeeding and late commencement of oral hygiene practices to an increased risk of caries [21]. Although the present study demonstrates an association between prolonged breastfeeding and the occurrence of dental caries, this relationship should be interpreted with careful consideration. It is essential to evaluate this association in the context of potential confounding variables, including suboptimal oral hygiene practices and nighttime feeding during the breastfeeding period.

In our study DMFT scores were significantly elevated among children lacking a personal toothbrush, those who did not engage in regular toothbrushing, and those with infrequent milk consumption. Similarly, children who had not received fluoride varnish exhibited higher DMFT scores. These results suggest that insufficient oral hygiene practices—especially before bedtime—and less-than-optimal dietary habits may substantially contribute to the development of dental caries in children. Kitsaras et al. [22] reported that 52.4% of parents indicated their children brushed their teeth nightly, with most children exhibiting DMFT scores greater than zero. Statistically significant relationships were identified between toothbrushing frequency, bedtime snacking/drinking habits, and DMFT scores. Moreover, a large-scale study of 2,000 adults aged 15 to 40 years revealed that individuals who did not brush their teeth, use dental floss, or use mouthwash experienced poorer DMFT outcomes [23]. A study by Aren et al. in Türkiye indicated that children with diets high in rice, pasta, and fruit juice presented significantly elevated mean DMFT scores. Conversely, daily meat consumption was associated with significantly lower DMFT scores. Furthermore, the intake of carbohydrate-dense snacks like biscuits and wafers between meals correlated with increased DMFT averages. These results underscore that frequent consumption of foods high in sugar is a notable risk factor for dental caries [18]. Overall, these findings highlight the multifactorial nature of dental caries and emphasize the necessity of integrating proper oral hygiene, balanced nutrition, and preventive care into children’s daily routines to achieve sustainable oral health outcomes.

Our study found that low socioeconomic status was associated with an increased prevalence of dental caries in children, whereas higher levels of maternal education were associated with a reduction in caries experience. Conversely, a higher level of maternal health literacy appeared to have an adverse effect on dental caries outcomes. Given the substantial proportion of school-aged children affected by dental caries, the findings underscore the necessity of early screening initiatives and parent-centered oral health education programs [24]. Şahin et al. [25]conducted a study assessing the impact of educational level on oral health using the CPITN and DMFT indices. The findings demonstrated that maternal education level significantly influenced oral health outcomes, highlighting the role of parental education—particularly that of mothers—in shaping children’s oral health status. Aren et al. [18]found that children from low-income families had relatively higher mean DMFT scores, suggesting a potential link between socioeconomic status and oral health outcomes. Research on the correlation between education and oral health in adults revealed no significant association between oral health literacy levels and either DMFT indices or Oral Health-Related Quality of Life scores [26]. Another study involving adults indicated that larger household sizes correlated with poorer oral and dental health outcomes. Comparative analyses of oral health indicator indices across various groups revealed a significant association between the DMFT index and individual education level, parental education, and socioeconomic status. Higher educational attainment was linked to lower mean DMFT scores, reinforcing the protective role of education in oral health [23]. Alraqiq et al. [27] conducted a study in Libya, which identified socioeconomic variables, such as maternal employment, and behavioral factors, like toothbrushing duration, as key factors associated with caries prevalence in children. The current investigation revealed average DMFT scores of 8.1 for children and 6.9 for their mothers. A weak but statistically significant positive correlation was found between the DMFT scores of mothers and their children. Additionally, a significant association was observed between increases in the child’s age and decreases in breastfeeding duration, as well as in maternal food literacy and health literacy scores. A cross-sectional study in Jordan, involving 264 children at a pediatric dental clinic, reported mean DMFT scores of 8.84 ± 5.39 for mothers and 6.17 ± 4.82 for children. While maternal age did not significantly correlate with the child’s DMFT score, a moderate, statistically significant correlation was identified between maternal and child DMFT scores. Furthermore, children from low- and middle-income families exhibited higher DMFT scores compared to those from high-income households [28].

In our study, while no significant relationship was found between mothers’ food literacy scores and children’s DMFT scores, higher maternal health literacy scores emerged as significant predictors of increased DMFT scores in their offspring. Although no previous study has directly examined the relationship between mothers’ health and food literacy levels and children’s dental caries, existing literature suggests that maternal behaviors and oral health literacy influence children’s caries experience. Among studies comparing children’s oral health outcomes at different stages of tooth eruption with parental oral health literacy scores, Vann Jr. et al. [29] reported that caregivers with lower oral health literacy scores had poorer oral health outcomes in children with a median age of 15 months. Similarly, Khodadadi et al. [30], in a study including children aged 21–84 months, found that those with inadequate parental Oral Health Literacy scores were at higher risk for dental caries. The relationship between high maternal health literacy and higher DMFT scores can primarily be explained by the structural characteristics of the DMFT index and differences in access to dental care services. DMFT is a cumulative indicator that includes not only active caries (“D”) but also treated (“F”) and missing (“M”) teeth. Mothers with high health literacy are more likely to detect their children’s cavities at an early stage and seek treatment, which may increase the “F” component. Therefore, a higher DMFT score may reflect early diagnosis and treatment access rather than poor oral health. In contrast, in the low health literacy group, caries may be detected later or remain untreated. Indeed, Yazdani et al. [31], in a study including children aged 5–15 years, found that those whose parents had adequate Oral Health Literacy had significantly more fillings and fewer missing teeth. As can be seen, even across different age groups, parents with low oral health literacy struggle to prevent caries and, when problems arise, tend to delay or fail to seek treatment or choose preventive approaches. This situation often leads to preventable decay resulting in tooth loss. Furthermore, the fact that our study was conducted among individuals attending a dental clinic may also have influenced the observed relationship between higher maternal health literacy and higher DMFT scores. Increased health literacy may positively affect mothers’ tendency to take their children to the dentist, thereby making dental problems more likely to be detected. Conversely, parents with lower health literacy might refrain from taking their children to the dentist altogether, resulting in undiagnosed cavities and an artificially lower DMFT score. The effect of this factor should be further clarified in larger, community-based studies. Moreover, interventions exclusively focused on enhancing mothers’ health knowledge may yield limited or negligible improvements in children’s oral health. It is therefore essential to address maternal behaviors and oral health literacy as pivotal components of oral health promotion strategies. Future literacy interventions should ensure that the concept of literacy extends beyond passive information dissemination and becomes a way of life. Practical, parent-oriented activities such as recognizing early signs of tooth decay and understanding treatment options can have a tangible impact on children’s filling and tooth loss rates. Indeed, a study examining the effects of maternal oral health literacy and mothers’ knowledge, attitudes, and practices on children’s dental health found that maternal behaviors and oral health literacy significantly affect children’s oral health, emphasizing that improving these maternal factors is key to enhancing children’s oral health outcomes [32].

This study has several limitations. Due to its cross-sectional design, a causal relationship cannot be fully established. Secondly, the findings may have limited generalizability due to the relatively small sample size and the single-center nature of the study. Furthermore, critical data such as breastfeeding duration and brushing habits were based on parental reports, which may introduce recall and reporting bias. In addition, caries experience was assessed using a combined index (dmft + DMFT) to capture both primary and permanent dentitions. Although this approach is commonly used in studies involving mixed dentition, potential variations in tooth eruption across the age range (40–120 months) may have influenced the comparability of scores between participants. Finally, unmeasured confounding factors that could influence dental caries, such as nighttime breastfeeding, could not be controlled, requiring cautious interpretation of the results.

## Conclusions

This study highlights the multifactorial influence of maternal and familial determinants on pediatric oral health, particularly dental caries. The findings demonstrate that lower parental education, maternal unemployment, and reduced socioeconomic status are significantly associated with higher Decayed, Missing, and Filled Teeth (DMFT) scores in children. Although maternal food literacy was not directly correlated with children’s DMFT scores, higher maternal health literacy emerged as a significant predictor of increased DMFT, while behavioral factors such as tooth brushing frequency, fluoride use, and feeding practices also showed notable effects. The modest positive correlation between maternal and child DMFT scores underscores the intergenerational nature of oral health behaviors. These results suggest that public health interventions should integrate literacy enhancement with behavior modification strategies to achieve comprehensive improvements in pediatric oral health, especially among socioeconomically disadvantaged groups. The implementation of early oral health education and routine screening programs targeting both children and parents is essential to reduce the burden of dental caries. Future longitudinal and cross-cultural studies are needed to establish causal relationships and further elucidate complex factors such as the association between prolonged breastfeeding and caries development, while controlling for potential confounders. Additionally, examining intrafamilial psychosocial dynamics may provide deeper insight into the underlying determinants of oral health disparities.

## Data Availability

Data will be shared upon request and with the consensus of the authors.

## Abbreviations

DMFT: Decayed, Missing, Filled Teeth
HLS-EU: European Health Literacy Survey Questionnaire
BMI: Body Mass Index
WHO: World Health Organization
SES: Socioeconomic status
SPSS: Statistical Package for the Social Sciences
